# Implementation of Motivational Interviewing Training in Dental Education and Research—A Scoping Review

**DOI:** 10.1002/jdd.13909

**Published:** 2025-04-27

**Authors:** Akash Ramprasad, Ayushi Naik, Nora Makansi

**Affiliations:** ^1^ Faculty of Dental Medicine and Oral Health Sciences McGill University Montreal Quebec Canada

## Abstract

**Purpose/Objectives:**

Motivational interviewing (MI) is an evidence‐based counseling approach that enhances behavior change by strengthening an individual's motivation. While MI research in dental education is growing, the scope of MI training programs has not been systematically examined. This review assesses the implementation of MI training in dentistry.

**Methodology:**

A scoping review followed the PRISMA‐ScR checklist and JBI manual for evidence synthesis. A systematic search (May 2023) using MeSH terms, keywords, and operators was conducted in Medline (Ovid), Embase (Ovid), CINAHL, PsycINFO (Ovid), Web of Science, and ProQuest. Articles were screened via Covidence, and data were extracted based on variables informed by the DoCTRINE framework.

**Results:**

Of 17 studies, 70.6% were US‐based, and 64.7% were published after 2010. MI training targeted dental hygiene and dentistry students (47% each) through lectures, role‐play, e‐learning, and workshops. Evaluations used pre–post questionnaires, recorded interactions, and tools like MITI, MISC, and OSCE. Training duration varied from single sessions (11.7%) to longitudinal programs (82.3%), enhancing students’ confidence, communication, and public health readiness. However, skill retention required ongoing reinforcement. Barriers included time constraints, patient resistance, and limited faculty support. High‐quality reporting (DoCTRINE score) was observed in 47% of studies.

**Conclusion:**

MI training enhances dental students’ communication and patient‐centered care. Long‐term skill retention requires continued practice, and hybrid instructional strategies can improve scalability. Faculty competency and structured longitudinal training are crucial for effective MI implementation.

## 1 Introduction

Some of the challenges oral healthcare professionals may face in providing care to their patients relate to supporting them in modifying long‐standing behaviors that may negatively affect oral health outcomes, the adoption and maintenance of health‐promoting behaviors, and compliance with treatment recommendations [1, 2].

Motivational interviewing (MI) is an evidence‐based communication approach that operates from four perspectives on practice: Partnership, Acceptance, Compassion, and Empowerment (PACE) [3, 4]. MI uses collaborative conversations to resolve ambivalence about change by strengthening the person's own motivation and commitment to the desired change [3].

Developed by psychologists William R. Miller and Stephen Rollnick in the 1980s, MI was initially used for counseling on alcohol addiction and substance abuse [5]. It is an evidence‐based approach that has been effectively applied by professionals in psychology, social work, medicine, and dentistry [6]. MI is compatible with the values of a wide range of disciplines and has been effectively used in areas like diabetes management [7], vaccine hesitancy [8], childhood obesity [9], and improving treatment adherence [10].

MI has four fundamental processes that describe the flow of “change talk”, mainly: engaging, focusing, evoking, and planning [11]. Change talk refers to the patient's own statements that may reveal motivation for, consideration of or commitment to change [3]. MI uses communication skills known as OARS to help address the voice of change: [11] Open‐ended questions, Affirmations, Reflective listening, and Summaries are the core skills used by providers to move the MI process forward and build a trusting relationship with the patient to discuss change.

In the context of oral health behavior counseling, MI training has been theorized and put into practice in multiple formats. Koerber et al. studied the effects of teaching brief motivational interviewing (BMI) to dental students for smoking‐cessation counseling [12]; Hinz et al. evaluated the impact of brief training of MI techniques within the dental school curriculum [13]; and several studies have been conducted to evaluate the impact of MI training in dental hygiene practice [14, 15, 16]. The literature demonstrates that MI is a feasible and efficient tool compared to traditional oral healthcare education approaches. However, the extent of implementation of MI training in dentistry, including optimal duration, structure of sessions, and evaluation of training outcomes, remains unclear [4, 13, 14, 16].

Therefore, this review aims to report the scope of MI training in dental/dental hygiene educational programs; examine the structure and characteristics of existing MI curricula; and assess the quality of reporting on MI curricula using the Defined Criteria to Report Innovation in Education (DoCTRINE) score.

## 2 Methods

This scoping review was reported in accordance with the Preferred Reporting Items for Systematic Reviews and Meta‐Analyses Extension for Scoping Reviews (PRISMA‐ScR) standards [17]. JBI's updated guidance [18] which is meant to be used in tandem with PRISMA‐ScR was used and is divided into nine steps (Appendix).

### 2.1 Search Strategy

**FIGURE 1:**
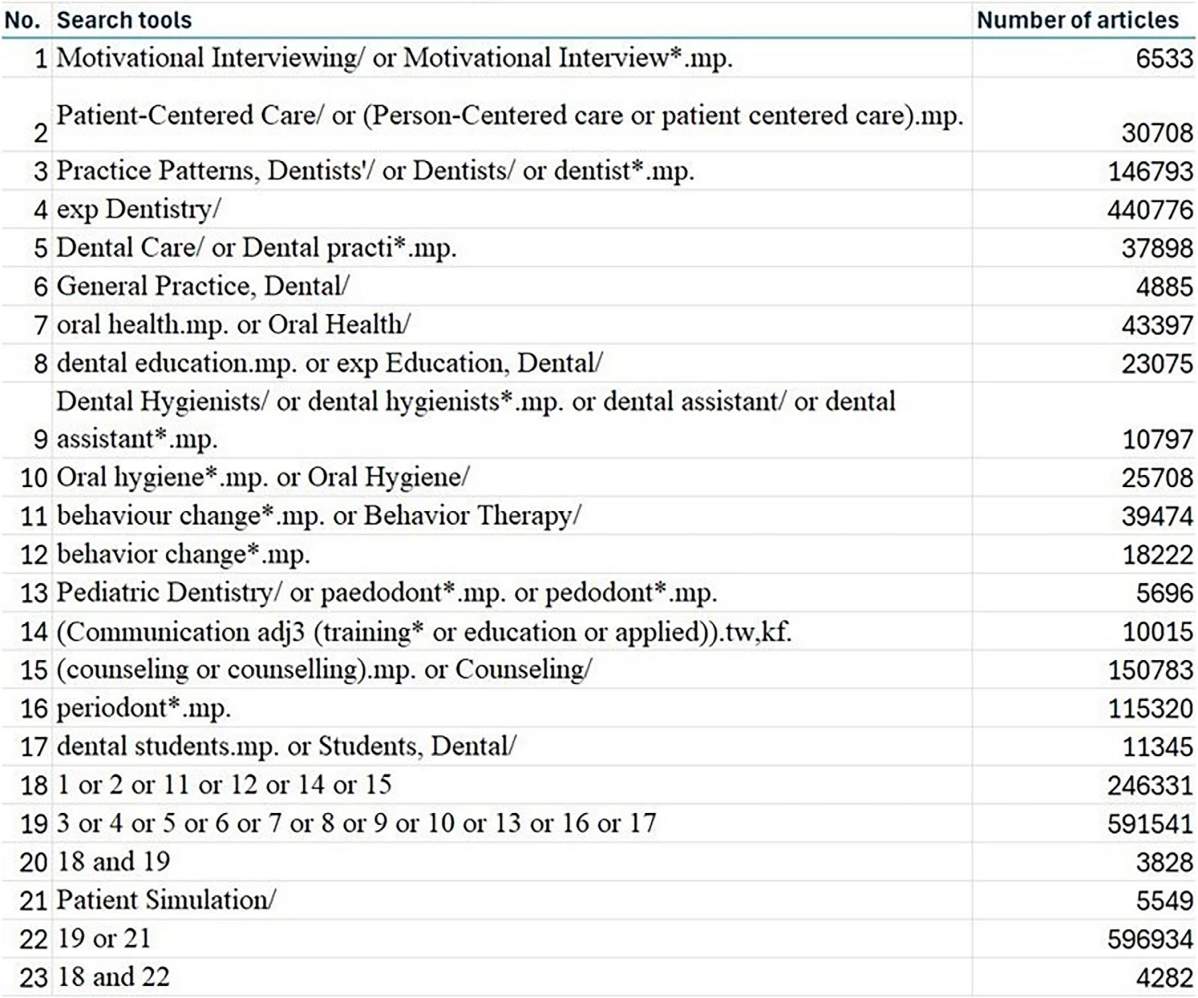
Search strategy.

The search strategy (Figure 1) was developed in collaboration with a McGill University librarian (JB) for the Medline (Ovid) database following which it was translated and adapted to Embase (Ovid), CINAHL, PsycINFO (Ovid), Web of Science and ProQuest. Search tools like Medical Subject Headings (MeSH terms), keywords, truncations and proximity operators were used to search the databases. The search terms used, and the number of articles retrieved are illustrated in Figure 1.

The search strategy was first implemented on May 31st, 2023. Then, a top‐search was executed to add any new published articles on 12th June 2024.

### 2.2 Study Selection and Appraisal

A. R. and A. N. independently screened the title and abstract of 50 unique titles, with an inter‐rater reliability of 98%, which was calculated as the percentage agreement. Any discrepancies were discussed with the research supervisor (N. M.), and the remaining screening was divided between A. R. and A. N. Next, the researchers completed the full review by independently reviewing each of the retained papers. Any discrepancies were discussed among the reviewers and in consultation with the supervisor. Criteria for inclusion were: 1) MI within dental education; 2) trainees in dentistry, dental hygiene, or dental assistance; 3) cross‐sectional studies, case‐control studies, randomized clinical trials, cohort studies, case series, case reports, all types of reviews, theses, and dissertations; 4) publications dating from 1980 onwards (MI was introduced in 1980). Exclusion criteria were: 1) non‐MI specific behavior counseling training; 2) letters, commentaries, conference abstracts, editorials, and duplicate studies.

### 2.3 Extraction and Synthesis

**FIGURE 2:**
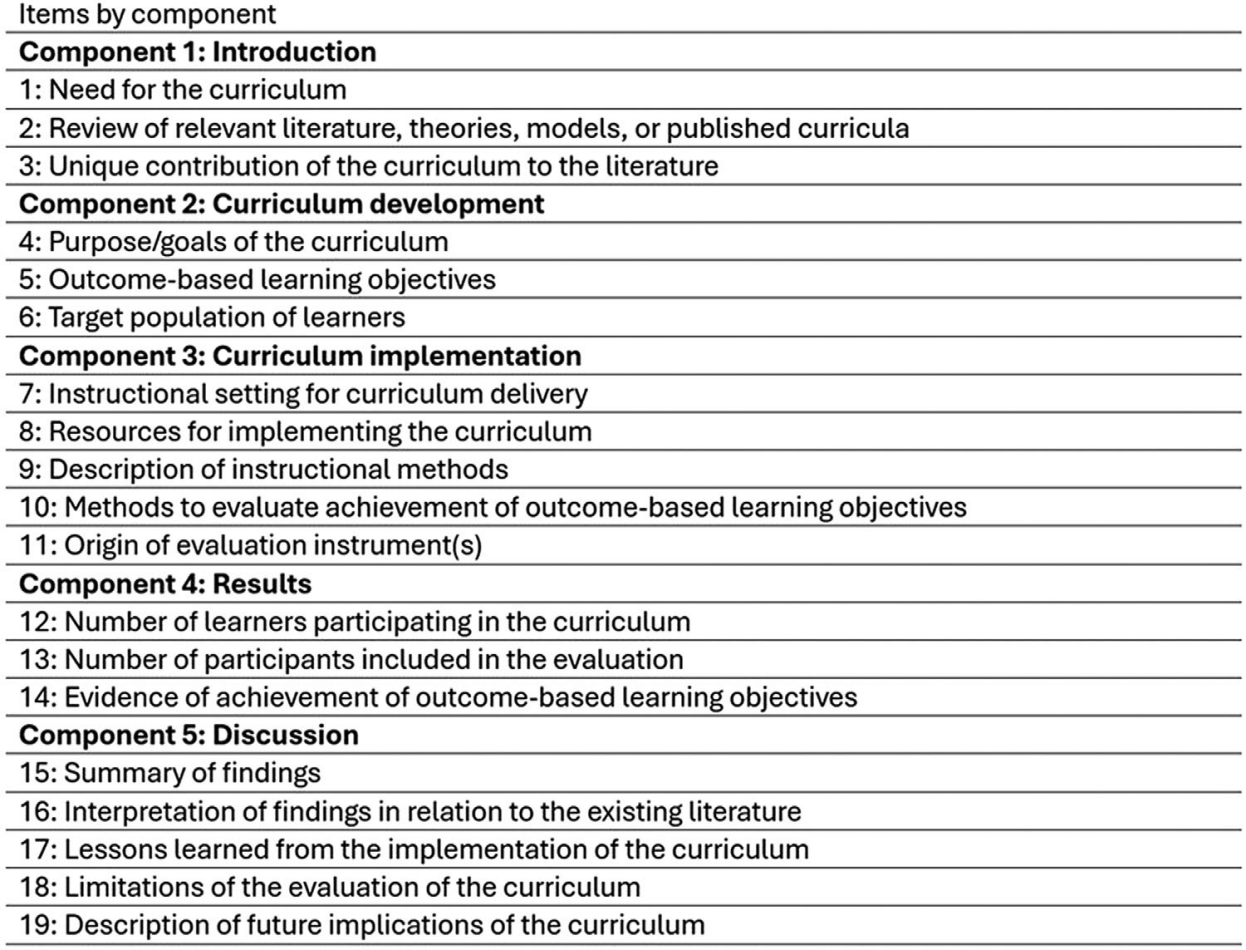
Defined Criteria to Report INnovation in Education (DoCTRINE) elements. Each of the 19 elements is scored dichotomously, using 1 = “present” or 0 = “absent”.

Variables were extracted according to Table 1 and Table 2. The use of a scoring system was employed: DoCTRINE [19]. The DoCTRINE score reflects the presence or absence of key components in reporting of new curricula (Figure 2). AR and AN divided the retained papers for individual extraction with regular consensus meetings, followed by cross checking extractions and discussing any discrepancies with the research supervisor. Authors cross‐checked extractions with a final meeting to discuss discrepancies.

**TABLE 1:** Summary of article extraction on MI training implementation.

Author Name, Year	Learner's profile	Profile of MI trainer (number)	Session frequency	Session duration	Instructional methods	MI training content
Arnett, 2022	Dental hygiene students + dual degree dental hygiene/dental therapy students	HPV faculty expert and MI trained DH educator (2)	Two sessions	90 min	BMI coaching role play	BMI strategies, OARS and EPE
Arnett, 2022	Dental hygiene students	HPV faculty expert, MI trained DH educator (2)	Control group: three in‐person sessions + 1 BMI coaching session Intervention group: same as above + additional MI refresher and online BMI coaching session	In‐person‐total duration:1 h 40 min BMI coaching: 50 min/session MI refresher: 40 min Online BMI coaching: 120 min	Lectures coaching role‐play	BMI strategies, spirit of MI, OARS, EPE
Arnett, 2017	Dental hygiene faculty members	UMKC MI experienced faculty members (NR)	Two sessions + several feedback sessions	Session 1: 14 h (2012) Session 2: 5 h (2014), grading and feedback sessions: (90 min each) in all semesters	Workshops grading and feedback	MI strategies, spirit of MI, OARS, EPE
Bray, 2021	Dental hygiene students	NR	Eight sessions for intervention group (over 1 year)	2 h per session	Didactic lectures 3 audio recordings with coaching feedback Ongoing support	MI strategies, MI principles, spirit of MI, OARS
Bray, 2013	Dental hygiene faculty members and students	Trainer 1: DH program director with MI counselor with formal MI training (Psychology department) (2); Trainer 2: DH clinic supervisors (NR)	Faculty: One session Students: Two sessions All: multiple follow up feedback sessions	Faculty: 1.5 days Students: 7 h	BMI coaching and practice workshops Role play‐assessment of audio recorded patient‐interactions	MI strategies, spirit of MI, OARS, EPE
Calleja, 2019	Dentistry, nursing, psychology, counselling, physician assistant students	SBIRT trained dental faculty member (1)	"Multiple"	Not reported	Lectures, readings discussions, coaching videos and demonstrations, role‐play	MI basics, Skills and the change process, brief intervention and treatment referral.
Croffoot, 2010	Dental hygiene students	Registered dental hygienist trained in MI (1)	Two sessions One Individual coaching and feedback sessions	Total duration: 7 h Coaching and feedback: 30–40 min	Lectures, readings handouts communication exercises	MI principles, use of MI, OARS
Curry‐Chiu, 2015	Dental hygiene mi trained alumni primarily practicing full‐time in general dentistry	Trained faculty members (NR)	Not reported	Total duration: 14 h	Didactic lectures, written exam, simulated patient exams, experiential practice activities, audio recordings with coaching feedback, ongoing support	MI strategies, MI principles, spirit of MI, OARS
Curtin, 2014	Dental clinicians and dental hygiene tutors	NR	One workshop	2 days	Not reported	MI strategies, MI principles, spirit of MI, OARS
Faustino‐Silva, 2019	Dentist and oral health technicians (OHT)	Staff member with PhD in psychiatry and extensive experience in conducting MI training (1)	Two sessions (2012) + 1 reinforcement training (2014)	4 h each session (2012) + 1‐h (2014)	Experiential format	MI strategies MI principles, OARS
Fuhrmann, 2021	Dental students (3rd and 4th year)	NR	Three sessions	1st session: 10 e‐lectures (15 min each), 2nd session: simulated interviews using MI in video form, 3rd session: apply MI techniques on real patients.	E‐lectures, videos recordings, web based trainings (WBTs)	Basics of MI, directive communication and MI
Hinz, 2010	Dental students (3rd year)	Not reported in the methods	Two sessions	1st session: 18 h of training, 2nd session: 3 h of lectures	Lectures, role‐plays.	Goals of MI, spirit of MI
Koerber, 2003	Dental students	Licensed clinical psychologist certified to teach motivational interviewing (MI) by MINT (1)	1 for control, 4 for Interventional	1st session: 3–4 h pretraining (common for all) 2nd–4th session: BMI training.	Seminars	Principles of MI, doing BMI in 5–10 min
Mills, 2017	Dental hygiene students (1st year)	Faculty members, expert in MI (NR)	Two sessions	1st session: 10 consecutive 50‐min sessions of enhanced MI learning, 2nd session: 110‐min class session on MI.	Readings, videos, presentation	Stages of change and health belief models, application of MI during patient care, strategies of MI, OARS, change talk
Schoonheim‐Klein, 2012	Dental students (3rd year)	Periodontics faculty member and a clinical psychologist trained in MI (2)	Control: 1, Interventional: Three sessions	M1: 4 h MI workshop, 1 h role‐play. M2: 4 h MI workshop, 3‐h role‐play. M3: 4 h MI workshop, 3‐h role‐play (more intensive)	Lectures, workshop, demonstrations and role‐play	Theories of MI, stages of change, essentials of MI
Stull, 2021	Dual degree dental hygiene/dental therapy students	Not Reported	One session	90 min	Role‐play	Using BMI techniques for HPV communication strategies.
Woelber, 2016	Dental students	Psychologist specialized in MI and psychiatrist specialized in MI. (2)	Two sessions	Workshop for 8 h, group supervision with psychiatrist for 4 h.	Workshop, readings	General behavior change, Understanding MI and it's abilities

**TABLE 2:** Summary of article extraction on Evaluation findings.

Author Name, Year	Country	Study type	Study design, description	Summary of findings	DoCTRINE score
Arnett, 2022	United States	Mixed methods	Quant: pre–post questionnaires and delayed post (UMN standardized MI rubric and open‐ended questions) Qual: analysis of 2 audio‐recorder patient interactions	1) Student perception and confidence increased from pre to post training PT1 but declined in PT2. 2) Improvement in faculty evaluation from 1st to 2nd patient interaction (PI) 3) BMI training insufficient to retain long‐term confidence.	18
Arnett, 2022	United States	Mixed methods	Quant: case‐control design and post‐evaluation using UMN Standardized MI rubric, OSCE with patients, and Kirkpatrick model. Qual: analysis of audio‐recorded HPV patient interaction.	1) 2nd Cohort received higher ratings compared to 1st cohort, except for the acceptance component of PI. 2) Both groups improved from PI1 to PI2 and OSCE. 3) ≥ 70% agreement in self‐assessment and faculty evaluation for 2 and 3 MI components for cohorts 1 and 2 respectively. 4) Skill‐based BMI training improved competence in MI and can be evaluated using the Kirkpatrick model.	18
Arnett, 2017	United States	Mixed methods	Quant: pre–post questionnaires qual: self‐assessment and team grading of student–patient MI interaction using MITI.	1) Despite the confidence, faculty members' ratings for the spirit of MI increased from pre‐test to PT1 but decreased by PT2. 2) Workshop benefited by improving faculty member's perception and confidence in teaching MI. 3) Both decreased towards the end of the year. 4) Team grading enhanced perception and teaching MI.	19
Bray, 2021	United States	Mixed methods	Quant: Case control, Multifaceted program evaluation, Qual: Analysis of audio recorded standardized patient interaction using MITI coding	1) Students trained in MI exceeded beginning proficiency and scored higher than the control group in all MITI indexes except for “complex reflections” 2) MI curriculum enhanced communication skills and competence for all the students but additional training might be needed	19
Bray, 2013	United States	Mixed methods	Quant: pre–post questionnaires*, final exam scores(students), qual: self‐assessment of simulated patient interaction. Audio recorded patient interaction. *Additional questionnaires (students): HCCQ, TSRQ	1) Faculty training showed increased perceived importance and confidence in MI strategies. 2) Knowledge acquisition was slightly higher for asynchronously delivered training, but no difference was found statistically compared to traditional. 3) BMI‐trained students showed slightly decreased motivation and confidence in poor oral health counseling but demonstrated significantly better patient engagement skills compared to those trained traditionally. 4) MI curriculum is effective and can enhance skills.	19
Calleja, 2019	United States	Quantitative	Quant: pre–Post evaluation, questionnaires (attitude on substance use)	1) Dental students demonstrated the greatest change in attitude toward substance abuse from pre to post‐SBIRT training and believed that all medical professionals should receive substance use counselling training. 2) Attitude change was more significant among females and black trainees whose belief change from not believing to considering it public health issue.	19
Croffoot, 2010	United States	Quantitative	Quant: pre–post evaluation and feedback, Qual: analysis of audio recorded patient interaction using MITI and MISC	1) Increased MI adherence through increased change talk and decreased closed questions among students. 2) MI adherence and MI non–adherence was related to the length of the session. 3) Mean and SD of summary scores of MITI and MISC indicated MI supportive behaviors. 4) DH students showed improvements in acquiring MI skills following a feedback/coaching session.	18
Curry‐Chiu, 2015	United States	Qualitative	Qual: case study, thematic analysis of one‐on‐one interviews, triangulation analysis of observational field notes and practice setting description	1) All interviewees used MI in their practice to improve patient care and rapport but were not confident about their skills. 2) MI was a superior patient communication style and is beneficial in the dental curriculum was reported. 3) Hygienist reported difficulty in implementing MI with resistant patients and within certain work environments with time constraints.	16
Curtin, 2014	Ireland	Descriptive	Not reported	No formal results, authors reported key learning outcomes based on their experience of the workshop: "Fundamentally, the MI training has been implemented for dental clinicians at a postgraduate or professional level. We would suggest that there are positive outcomes in terms of learning potential for trainee dental clinicians at an undergraduate level."	9
Faustino‐Silva, 2019	Brazil	Quantitative	Quant: randomized community trial (longitudinal follow‐up), Pre and post evaluation and delayed post‐evaluation, questionnaires used: importance and confidence ruler for question", Qual: dialogue interview, and helpful response questionnaire	1) MI‐ knowledge prior to training was homogenous in tests (MI trained) and control groups (No‐MI training). 2) The test group showed significant improvements in using open questions, affirmations, and reflective listening, and decreased closed questions. 3) Both the Importance and Confidence of Rulers demonstrated substantial increases. 4)The skills acquired during the training were mainly maintained over one and two years. 5) Annual follow‐up meetings helped reinforce these skills, ensuring continued application of MI techniques.	19
Fuhrmann, 2021	Germany	Quantitative	Qual: pre–post evaluation with feedback and using questionnaires for analysis and MITI‐d for evaluation	1) The students demonstrated high MI‐adherent behavior, with performance comparable to traditional classroom settings. 2) Global scores for empathy and MI spirit indicated effective MI practice, and students successfully applied MI techniques in patient interactions. 3) Analysis of patient interviews showed adherence to MI principles, indicating effective skill transfer from e‐learning to clinical practice.	19
Hinz, 2010	United States	Qualitative	Qual: pre–post evaluation followed by grading based on accurate usage of MI terms.	1) Most students reported a positive experience using MI with the added benefit of being brief. 2) It helps them better plan their communications with their patients.	17
Koerber, 2003	United States	Quantitative	Experimental pre–post evaluation with analyzing of recorded student interaction videotapes and questionnaires	1) Through the training, students showed significant improvement in their use of BMI techniques, with patient involvement playing a crucial role. 2) The interventions that enabled patient transformation helped establish rapport.	15
Mills, 2017	United States	Quantitative	Retrospective pre–post evaluation, analysis of audio recorded role‐play assignments and questionnaires.	1) The enriched curriculum, which provided opportunities for repeated practice, feedback, and the practical application of MI in clinical settings, greatly increased the significance and confidence levels of MI among dental hygiene students. 2) Consistent reinforcement and early exposure to MI throughout the curriculum were recognized as crucial for developing these skills. 3) This study advocates for integrating MI training into dental hygiene education to improve patient‐centered communication and behavior‐change counselling abilities.	19
Schoonheim‐Klein, 2012	Netherlands	Quantitative	Pre–post evaluation with questionnaire, formative assessment (OSCE), questions from a validated questionnaire developed by Allard (22) for evaluation of dentists’ attitudes towards smoking.	1) The group of students who received the most extensive motivational interviewing (MI) education, followed by a formative assessment, experienced a significant reduction in smokers among patients and students. 2) As a result of MI education, the student's understanding of the connection between smoking and periodontitis increased from 33% without MI to over 96% in the groups with MI. 3) Educating dental students in MI positively impacted the percentage of periodontal patients and students who quit smoking. 4) Therefore, involving dental students in smoking cessation with MI shows promise when integrated into periodontal education.	15
Stull, 2021	United States	Quantitative	Retrospective pre–post evaluation with feedback and analysis of audio recorded patient interaction sessions.	1) The results indicated that the intervention and control groups experienced improved knowledge immediately after completing an online educational module. 2) However, there was a slight decline in knowledge after practical patient interactions, suggesting the importance of holding repeated educational sessions to sustain knowledge levels. 3) Over time and with faculty feedback, confidence in motivational interviewing (MI) skills increased. 4) Although students initially displayed overconfidence, real patient interactions highlighted challenges such as time management and securing patient consent for discussions.	19
Woelber, 2016	Germany	Quantitative	pre–post evaluation with questionnaires and analysis of audio recorded patient interactions using MITI‐d.	1) MI significantly enhanced the quality of behavior‐related discussions. Compared to the control group, participants in the MI group demonstrated higher levels of empathy, MI spirit, and MI‐adherent communication. 2) The MI group's performance was characterized by a greater use of open‐ended questions, complex reflections, and a more favorable ratio of reflections to questions. 3) These specific enhancements in communication skills are consistent with the strategies employed in MI. 4) Despite these improvements in communication, the duration of behavior‐related discussions and the amount of information provided by the students did not show significant differences between the MI and control groups. 5) The substantial impact of scaling and root planning on clinical parameters outweighed any potential influence of MI within the study's timeframe.	15

Abbreviation: BMI, brief motivational interviewing; HCCQ, Health Care Climate questionnaire; HPV, human papilloma virus; MI, motivational interviewing; MISC, Motivational Interviewing Skill Code; MITI, Motivational Interviewing Treatment Integrity; NR, not reported; OSCE, Objective Standardized Clinical Examination; PT, post training; SBIRT, screening, Brief Intervention, and Referral to Treatment; TSRQ, Treatment Self‐Regulation Questionnaire; UMN, University of Minnesota.

## 3 Results

**FIGURE 3:**
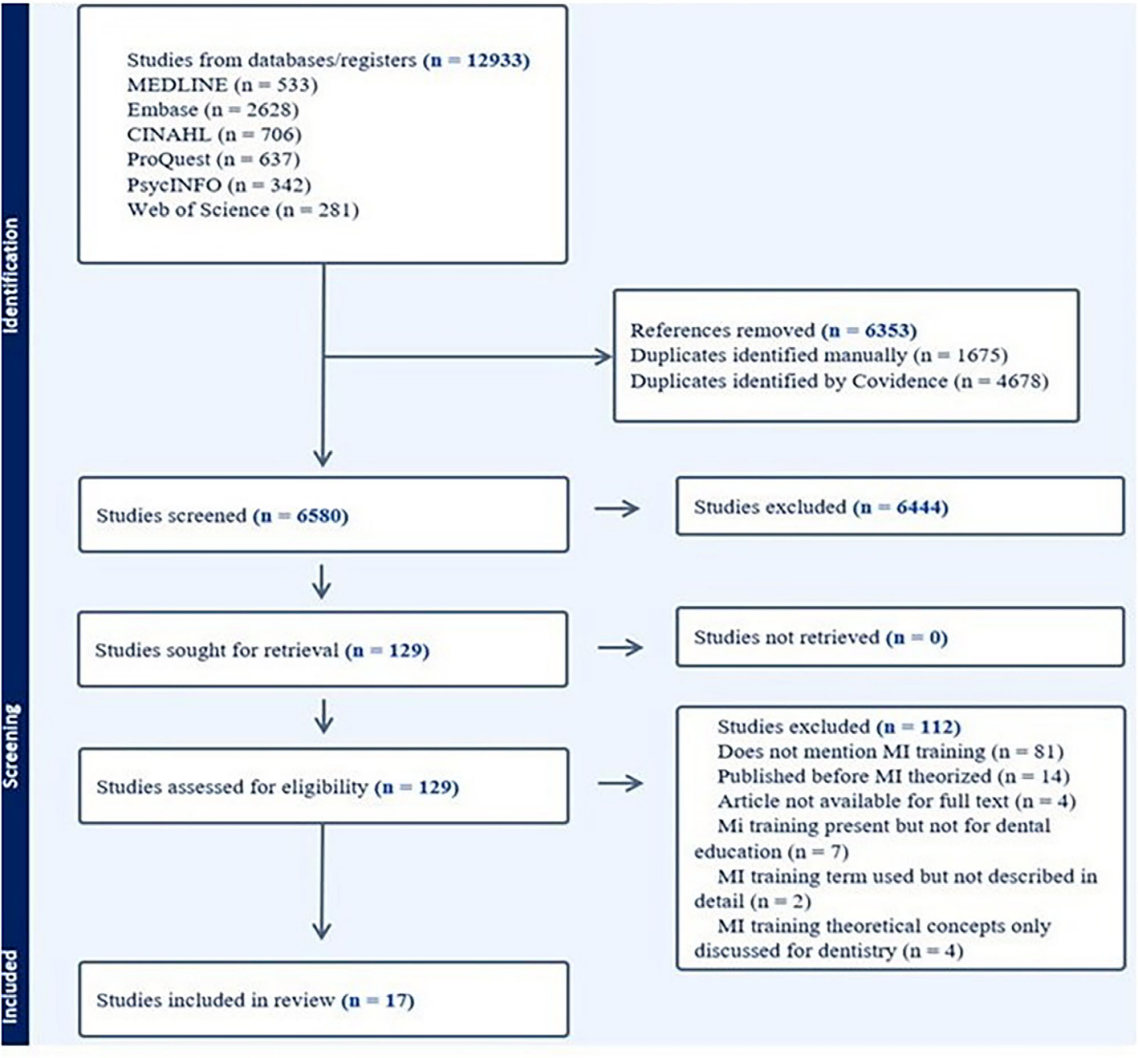
PRISMA flowchart.

A total of 17 papers (13.1%) were reviewed. A PRISMA flow chart (Figure 3) illustrates the detailed study selection process. Most of the article (n = 16, 99%) were published 2010 onwards, and 12 out of 17 (70.58%) were conducted in the United States.

### 3.1 MI Instruction, Frequency, and Duration

Six of the reported MI (35.2) programs used didactic instruction methods [12, 14, 16, 20, 21, 22], five papers reported the use of applied training measures (29.4%) such as workshops, coaching/role‐play [15, 23, 24, 25, 26]. The remaining six papers (35.2%) incorporated both didactic as well as practical instructional methods [13, 27, 28, 29, 30, 31].

Furthermore, three papers (17.6) reported the use of e‐learning modalities like videos in their training [21, 22, 28]. Another three papers (17.6%) specified the strategy of providing feedback to the students to improve performance [14, 20, 24].

The reported frequency of the sessions was categorized into one‐time/single session and longitudinal/multiple sessions. A total of 14 papers (21.4%) reported a longitudinal format [12, 13, 15, 16, 20, 21, 22, 23, 24, 27, 28, 29, 30, 31]. Three papers (21.4%) out of 14 included a separate session dedicated to providing feedback to the students [15, 16, 24]. The duration of each session ranged from 30 min to 1.5 days. Two papers (11%) reported on one training session, their durations were 2‐days and 90 min, respectively [25, 26]. One paper (5.8%) did not report on training frequency [14].

### 3.2 MI Content

All the studies focused on teaching MI principles (expressing empathy, developing discrepancy, rolling with resistance, and supporting self‐efficacy) alongside OARS (Open‐ended questions, Affirmations, Reflective listening, and Summaries) and the EPE (Elicit‐Provide‐Elicit) approach. Four papers (23%) tailored their training around BMI [12, 23, 25, 27], a condensed version of MI for time constrained environments that aims to aid patients in their desire to change, while the remaining papers reported framing the content within the traditional MI framework.

### 3.3 MI Trainees and Trainers’ Profiles

As shown in Table 1, eight MI training programs (47%) were conducted with dental hygiene students [14–16, 20, 22, 23, 25, 27], Two of which also included alum participants and faculty instructors respectively [14, 24]. The remaining eight (47%) were conducted with undergraduate dental students [12, 13, 21, 26, 28–31].

Twelve papers (70.5%) described employing trainers who were specialized/experienced in MI [12, 14–16, 22–24, 27–31], with four out of the twelve reporting a background in psychology or psychiatry. The remaining five studies did not report the presence of an MI‐qualified trainer [13, 20, 21, 25, 26]. In terms of the number of trainers facilitating the sessions, three of the twelve studies (25%) had two trainers delivering the MI training [15, 29, 30]. The remaining nine studies reported that one trainer facilitated the MI training.

### 3.4 Evaluation Design

Different evaluation methodologies were used to assess the MI curricula: ten (58.8%) were quantitative in nature [12, 16, 21, 22, 25, 26, 28–31]; two (11%) were qualitative [13, 14]; and five (29.4%) used a mixed‐methods approach [15, 20, 23, 24, 27]. The predominant methods used to evaluate the MI training included self‐report pre–post questionnaires and the analysis of audio recordings of patient interactions.

In addition, specific tools were employed including: (1) MITI and MITI‐d to measure fidelity of MI (*n* = 5, 29.41%) [16, 20, 21, 24, 30]; (2) MISC to measure adherence to MI (*n* = 1, 5.88%) [16]; (3) OSCE to measure clinical competence (*n* = 2, 11.76%) [27, 29]; (4) Kirkpatrick model (*n* = 1, 5.88%) to measure MI competence [27]; and (5) HCCQ (Health Care Climate Questionnaire) and TSRQ (Treatment Self‐Regulation Questionnaire) questionnaires [15].

### 3.5 Evaluation Outcomes

In terms of impact on communication skills, one paper reported that students trained in BMI displayed enhanced patient engagement skills compared to those who were not [16]. A study conducted a few years later by the same author found that a comprehensive MI curriculum significantly improved the students’ communication skills and MI skills as rated by the most rigorous fidelity measure using the MITI [20]. Other findings included improvement in quality of discussions related to behavior and enhancement of communication skills (such as open‐ended questions and affirmations) [21, 22]. Moreover, a marked increase in empathy following MI training was also reported [21, 30].

In terms of developing and maintaining MI skills, Mills et al. found that dental hygiene students’ perceptions of the importance of MI and their confidence in delivering MI were enhanced over time [22]. Early exposure to MI training and consistent reinforcement were recognized as crucial elements in developing MI skills. Also, three papers found that MI programs that integrate feedback sessions regarding patient interactions have greater effectiveness from the perception of participants and trainers [16, 24, 25]. These studies showed that regular or frequent feedback sessions help enhance the students’ confidence and attitude towards delivering MI.

In accordance, Arnett et al. found that a single session of BMI training was insufficient to maintain long‐term confidence while delivering MI [23]. Another paper suggested students may need additional ongoing training to achieve competence in MI [^24^].

The most common limitations reported across majority of the studies were the presence of a small sample size and absence of a control group [12, 13, 16, 22–24, 27, 28]. Another reported limitation was the lack of trained faculty members to mentor clinical MI training [16].

The DoCTRINE score ranged from 9 to 19 (range 0–19 points), with an average score of 17, and median of 18. Each criterion is scored as “*Y*” (1 point) or “*N*” (0 points), with the total score reflecting how well educational innovation reporting adheres to the checklist's standards. At the end of the checklist the final score is calculated out of 19 by adding the scores of all the items.

A score of 9 displays poor reporting of data in the article while a score of 19 showcases excellent adherence to the rigorous standards in reporting.

## 4 Discussion

This scoping review assesses and summarizes the current literature on the implementation of MI training in dental education and research. The results of this scoping review indicate an increase in publications on MI training programs in dental education over the years. The research studies that were reviewed evaluated multiple formats and structures of MI curricula for oral health professionals. The findings highlight the importance of MI training in providing dental students with a solid foundation for applying MI in clinical practice.

Students who received MI training demonstrated greater proficiency in its application and adherence than those without training. Moreover, a few papers demonstrated a marked improvement in trainees ability to employ techniques like open‐ended questioning, affirmations, and reflective listening [23, 30]. The communication improvement through MI training translates to clinical practice as students report increased confidence in discussing sensitive topics and behavior counselling such as cancer diagnosis disclosure, smoking cessation, and oral hygiene [22, 29]. MI training effectively decreased smoking rates among periodontal patients, illustrating its potential in preventive care [29]. Similarly, Calleja et al. found that MI training shifted dental students' attitudes toward addressing substance abuse, with trainees recognizing the importance of integrating behavioral health counseling into dental care [28].

Furthermore, some of the papers showed an increase in empathy among MI‐trained students compared to those who did not receive the training [20, 21]. Studies have shown that stress, burnout and heavy workloads contribute to the decline of empathy among medical students and professionals over time [32]. An empathetic dentist–patient relationship also fosters trust, compliance, and overall patient satisfaction [33].

While most of the studies used multiple instructional methods such as didactic, role‐play, and simulated patient engagements to train and evaluate students. The e‐learning approach was only utilized by Fuhrmann et al. who showed that it can be effectively used in combination with simulated interviews and real‐patient interactions, especially in schools with limited in‐person training resources [21].

Apart from the positive influence of MI training, several studies identified structural and systemic barriers in MI implementation, including limited faculty expertise; time constraints during patient interactions; and difficulties adapting MI techniques to resistant patients or fast‐paced clinical environments [14, 25]. Development of the faculty's proficiency was seen to be crucial in overcoming these barriers, as the teachers' competency in MI greatly influences students' learning outcomes [15]. Time constraints during patient interactions limit the application of MI techniques, especially in clinical settings where efficiency is primarily considered as a priority. Training programs must address this challenge by teaching brief but effective MI interventions, as demonstrated by Koerber et al., where students learned to apply MI strategies within 5–10 min [12]. Research conducted by Mills et al. and Bray et al. [15, 22] supports the integration of MI training throughout the dental curriculum, ensuring that students encounter MI principles both early on and repeatedly.

This scoping review, to our knowledge, is the first evidence to date emphasizing and summarizing the implementation of MI training in dental education. Although the quality of reporting of included studies varies, majority of the studies had a relatively high DoCTRINE score, indicating thorough reporting on the implementation of MI training. Such practice informs future efforts to design and implement MI programs by providing detailed descriptions of existing programs.

One of the limitations to this scoping review is susceptibility to potential bias, primarily selection bias and publication bias, as studies with positive results are more prone to get published. Also, the retrieved studies were diverse in their study designs which increased the heterogeneity of the reviewed papers.

The current knowledge on MI training in dental education is limited, highlighting the need to develop and identify best practices for selecting and designing curricula that effectively incorporate MI. In addition, consistent use of validated MI evaluation measures would ensure quality in delivering of MI training. Further research is essential to determine the optimal duration and frequency of training required to enhance MI adherence and competence, ensuring long‐lasting effects in clinical practice. Effective strategies to train instructors in MI principles and techniques should be explored considering the impact of instructor's proficiency on student learning and performance. Longitudinal studies specifically on MI skills retention for more than one or two years are needed to identify effective reinforcement protocol.

Future studies can also compare the effectiveness of various instructional methods and MI training modalities like e‐learning and hybrid formats as well as evaluate the cost effectiveness of these methods. Considering the possible time constraints in clinical healthcare practice, more studies can focus on the development of a BMI protocol to be delivered in 10‐min patient interactions.

Currently, no studies have evaluated MI training and its outcomes through a multi‐institutional study. Therefore, such studies in the future might be able to provide insight into the most effective training protocols. Studies involving MI‐trained dental hygiene practitioners and clinicians are needed to further understand challenges in application of MI in routine practice and to know training effectiveness.

## 5 Conclusion

The findings from this scoping review emphasize the critical role of MI training in enhancing dental students' communication skills, empathy, and patient‐centered care abilities. In terms of implementation, longitudinal curricula and applied learning strategies were shown to reinforce MI principles. At the same time, hybrid models that incorporate e‐lectures and simulated interviews offer scalable training solutions, particularly in resource‐limited environments.

Despite challenges such as limited faculty expertise, time constraints, and variability in training methods, the findings show that MI training prepares students for real‐world patient interactions and helps them address common issues. To enhance the effectiveness of MI training, educational institutions must prioritize the development of expert faculty, adopt a consistent training framework, and ensure the availability of resources to facilitate ongoing practice and follow up. By addressing these limitations and sustaining MI training throughout the curriculum, dental schools can produce competent, empathetic, patient‐centered practitioners better equipped to support behavior change and improve oral health outcomes.

## Conflicts of Interest

The authors declare no conflicts of interest.

## 1

**Table:**

JBI'S updated guidance for scoping reviews
1) Defining and aligning the objective/s and question/s.
2) Developing and aligning the inclusion criteria with the objective/s and question/s.
3) Describing the planned approach to evidence searching, selection, data extraction, and presentation of the evidence.
4) Searching for the evidence
5) Selecting the evidence
6) Extracting the evidence
7) Analysis of the evidence
8) Presentation of the results
9) Summarizing the evidence in relation to the purpose of the review, making conclusions, and noting any implications of the findings.
